# Genome comparison without alignment using shortest unique substrings

**DOI:** 10.1186/1471-2105-6-123

**Published:** 2005-05-23

**Authors:** Bernhard Haubold, Nora Pierstorff, Friedrich Möller, Thomas Wiehe

**Affiliations:** 1 Department of Biotechnology & Bioinformatics, University of Applied Sciences, Weihenstephan, Germany; 2 Institute of Genetics, Universität zu Köln, Zülpicher Straße 47, 50674 Köln, Germany; 3 Berlin Center for Genome Based Bioinformatics and Freie Universität, Berlin, Germany

## Abstract

**Background:**

Sequence comparison by alignment is a fundamental tool of molecular biology. In this paper we show how a number of sequence comparison tasks, including the detection of unique genomic regions, can be accomplished efficiently without an alignment step. Our procedure for nucleotide sequence comparison is based on shortest unique substrings. These are substrings which occur only once within the sequence or set of sequences analysed and which cannot be further reduced in length without losing the property of uniqueness. Such substrings can be detected using generalized suffix trees.

**Results:**

We find that the shortest unique substrings in *Caenorhabditis elegans*, human and mouse are no longer than 11 bp in the autosomes of these organisms. In mouse and human these unique substrings are significantly clustered in upstream regions of known genes. Moreover, the probability of finding such short unique substrings in the genomes of human or mouse by chance is extremely small. We derive an analytical expression for the null distribution of shortest unique substrings, given the GC-content of the query sequences. Furthermore, we apply our method to rapidly detect unique genomic regions in the genome of *Staphylococcus aureus *strain MSSA476 compared to four other staphylococcal genomes.

**Conclusion:**

We combine a method to rapidly search for shortest unique substrings in DNA sequences and a derivation of their null distribution. We show that unique regions in an arbitrary sample of genomes can be efficiently detected with this method. The corresponding programs shustring (SHortest Unique subSTRING) and shulen are written in C and available at .

## Background

Sequence comparison is traditionally carried out using alignments. The alignment procedure ensures that only homologous positions are compared and corresponding algorithms form the classical core of bioinformatics [1-3]. Once a sequence alignment has been computed, it can be used to determine, for example, signature oligonucleotides or unique genomic regions among a group of closely related organisms.

Perhaps surprisingly, the applications of alignments just mentioned – signature oligos and detection of unique genomic regions – do not necessarily involve an alignment step. Since the computation of alignments tends to take time proportional to the product of the lengths of the sampled sequences, elimination of this step often leads to dramatic increases in the speed of sequence analysis algorithms [4].

**Figure 1:**
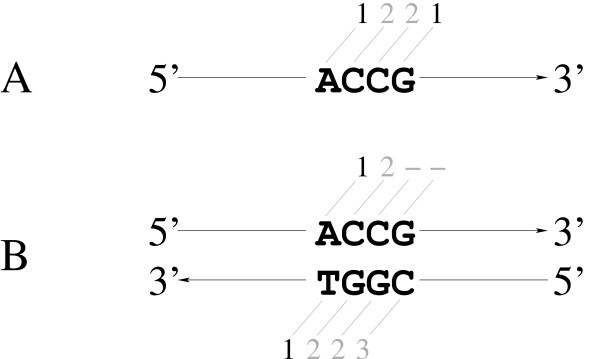
**Shustrings on forward and backward strands**. Global and local shortest unique substrings ('shustrings') in the DNA-sequence ACCG. **A**: The global shustrings are A and G, and have length 1 (black numbers above the sequence). The numbers above the sequence indicate the length of the four local shustrings A, CC, CG and G present on the forward strand. **B**: In the presence of the reverse strand global as well as local shustrings may change. For some positions at the 3'end of the sequence shustrings may not be defined (here, position 3 and 4 on the forward strand). Notice that the complement of a local shortest unique substring is also unique, however not necessarily a shortest unique substring (for example the pair GT on the reverse strand and AC on the forward strand). The complement of a global shortest unique substring is again a global shortest unique substring (here the two global shustrings A and T).

Our method of alignment-free sequence comparison is based on the idea of "shortest unique substrings", that is, the shortest substrings of a sequence which are not found elsewhere. Consider for example the sequence *S *= ACCG. It contains  substrings, of which the following eight are unique: {A, AC, ACC, ACCG, CC, CCG, CG, G}. Two of these are *shortest *unique substrings: {A, G}. Such *global *shortest unique substrings can occur anywhere in *S*. In contrast, we define *local *shortest unique substrings to be tied to a specific position in *S*. More formally, we determine for every position *i *in *S *the length *x *of the substring *S*[*i*..*i *+ *x *- 1] such that it is unique while *S*[*i*..*i *+ *x *- 2] is not. In the case of our example string, the result is *x *= 1 for the first position, *x *= 2 for the second, *x *= 2 for the third, and *x *= 1 for the last. Figure 1A gives a graphical representation of these local shortest unique substrings.

So far we have only considered the forward strand of a given DNA sequence. In the presence of the reverse strand, the set of *x*-values changes to *x *= 1 for the first and *x *= 2 for the second position. No well-defined unique substrings start at the third or the last position of the sequence. Figure 1B illustrates the shortest unique substrings found on the forward and reverse strands of our example sequence. It is important to realize that when dealing with double-stranded DNA, the set of shortest unique substrings is different from that found in single-stranded DNA. However, complementarity of DNA ensures that the complement of a local shortest unique substring is again a unique substring, though not necessarily a shortest unique substring (cf. Figure 1B). In contrast, the complement of a global shortest unique substring is also a global shortest unique substring.

Application of shortest unique substrings to biological problems requires both their efficient detection and knowledge of their probability distribution. The latter is derived in this paper. As to the detection of shortest unique substrings, a data structure known as the suffix tree can readily be used for this purpose [4]. We demonstrate the utility of shortest unique substrings for sequence analysis by applying them to three tasks: (i) identification of signature oligo nucleotide sequences, (ii) detection of unique as well as repeat regions in the genome of *Mycoplasma genitalium*, and (iii) detection of unique genomic regions in strain MSSA476 of the human pathogen *Staphylococcus aureus *when compared to four other staphylococcal genomes.

## Results

### Global shortest unique substrings in *Caenorhabditis elegans*, human, and mouse

The genome of *C. elegans *is one of the smallest metazoan genomes sequenced to date. It consists of five autosomes and one sex chromosome, amounting to 100.29 Mb of sequence information [5]. When searching for global shortest unique substrings in this genome, we found a single complementary pair of unique motifs of length 10 located on chromosome 1. Considering the next shortest unique substrings (length 11) we found a total of 10,509 such motif pairs distributed among the five chromosomes. Note that the search for these globally unique substrings was done with respect to the forward and backward strands of the complete genome.

We repeated this analysis for the human genome, which is the largest genome sequenced to date. It consists of 22 autosomes and two sex chromosomes totalling 2.84 Gb published sequence data [6]. We found 215 pairs of global shortest unique substrings of length 11 distributed on the autosomes and the X-chromosome. The Y chromosome contained no unique sequences of length 11 but 135 globally unique sequence pairs of length 12.

We were puzzled by the fact that – with the exception of the single instance of a unique substring pair of length 10 on chromosome 1 of *C. elegans *– the shortest unique substrings in humans had the same length (11) as those found in *C. elegans*, even though the human genome is 28 times larger than that of the nematode. When repeating the search for global shortest unique substrings in the mouse genome, whose size is similar to that of human (2.49 Gb), we found a matching result: there were 255 shortest unique substring pairs of length 11 distributed among the autosomes and the X-chromosome. On the Y-chromosome there were 38 unique substring pairs of length 12. The fact that the highly repetitive Y chromosome contained global unique substrings of length 12 as compared to length 11 on autosomes suggested that the length of shortest unique substrings is inversely related to genome information content. In order to further explore whether particularly short unique substrings are associated with functional regions of the genome, we investigated the distribution of globally shortest unique substrings among 1 kb upstream regions of annotated genes.

A total of 29 out of 2 × 350 shortest unique substrings were located among a non-redundant set of 16,286 human 1 kb upstream regions. The probability of observing a single hit in an upstream region with one shortest unique substring is equal to the fraction of the published genome (considering again forward and backward strands) covered by upstream regions. This is

*f*_h _= 16286 × 1000/(2 × 2.84 × 10^9^) ≈ 0.003.

The probability of observing 29 or more hits to upstream regions under the null hypothesis of equal distribution is



A similar result was obtained for the mouse genome. Here a total of 13,985 non-redundant upstream regions contained 22 of the 2 × 293 shortest unique substrings. The probability of finding a single hit with one shortest unique substring is again equal to the fraction of the published genome covered by the upstream regions:

*f*_m _= 13985 × 1000/(2 × 2.49 × 10^9^) ≈ 0.003.

The probability of observing 22 or more hits to upstream regions by chance is



In other words, both in the human as well as in the mouse genome shortest unique substrings are clustered close to genes. A complete list of the shortest unique substrings with hits to upstream regions is available as supplementary material (see Additional file 1).

So far we have concentrated on the overall, i.e. global, shortest unique substrings. In the following sections we extend this analysis to include all local shortest unique substrings.

### Empirical distribution of local shortest unique substring lengths

**Figure 2:**
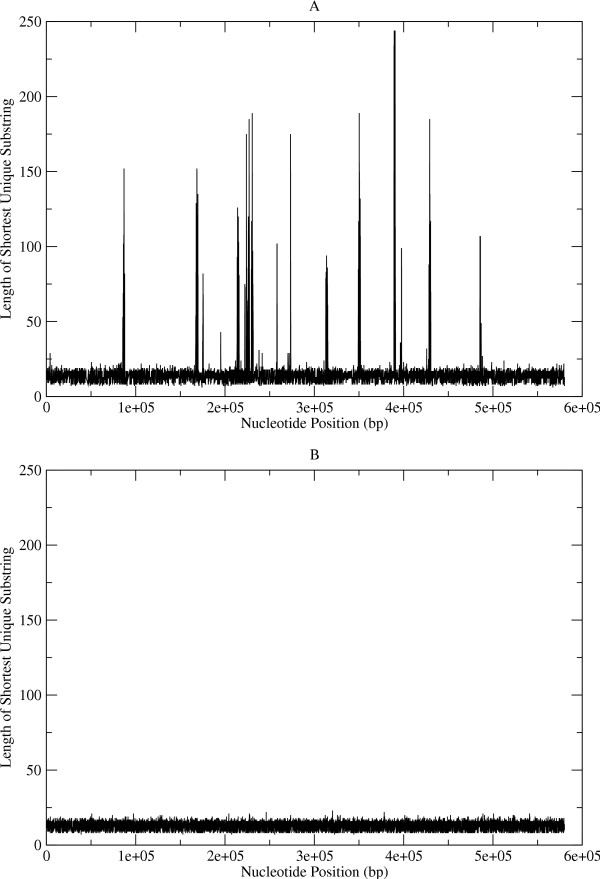
**Shustrings in *Mycoplasma genitalium***. **A**: Lengths of the local shortest unique substrings at every position along the genome of *Mycoplasma genitalium*. The lengths displayed minus one correspond to the lengths of substrings which are repeated at least once in the genome. **B**: The same as **A**, except that the nucleotides in the genome were shuffled (thus preserving nucleotide frequencies), which leads to the disappearance of long repeats.

The pathogenic bacterium *Mycoplasma genitalium *has one of the smallest genomes known for any free-living organism [11]. The 580,074 bp of its genome encode 480 open reading frames and would be expected to be void of redundant, that is repetitive, sequences. However, when plotting the length of all local shortest unique substrings contained in its genome, we detected 26 non-overlapping shortest unique sequences longer than 100 bp, the longest of which spanned 244 bp (Figure 2A). In other words, the genome of *M. genitalium *contains a perfectly conserved repeat sequence that is 244 - 1 = 243 bp long. The statistical significance of these repeats is illustrated in Figure 2B, which displays the lengths of the shortest unique substrings in a shuffled version of *M. genitalium*'s genome. In such a scrambled sequence devoid of biological meaning no shortest unique substring is longer than 21 bp. Having found surprisingly short, as well as surprisingly long shortest unique substrings, we proceed by deriving the null distribution of shortest unique substring lengths.

### Distribution of local shortest unique substring lengths

**Figure 3:**
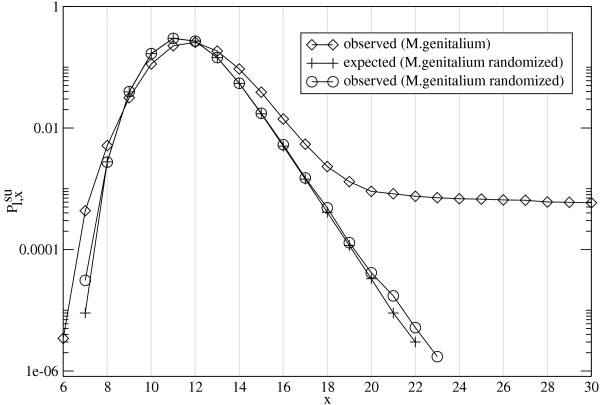
**Shustring probability distribution in the randomized genome of *Mycoplasma genitalium***. Observed and expected distributions of the lengths *x *of local shortest unique substrings. The "observed" distribution was obtained by shuffling the nucleotides of the genome of *Mycoplasma genitalium *(c. f. Figure 2), while the expected distribution is based on equation (1) using the genome's GC-content of 2*p *= 0.316 and length *l *= 580,074 bp.

As explained further in the Methods section, the probability of finding shortest unique substrings of length *x*, , is the number of unique substrings of length *x*, , minus the number of unique substrings of length *x *- 1, , divided by the genome length *l*:



where



and 2*p *represents the GC-content of the genome (*p *∈ [0, 1/2]). Figure 3 demonstrates that the fit between equation (1) and the empirical distribution of local shortest unique substrings (cf. Figure 2B) is excellent. Equation (1) provides an efficient method for establishing the statistical significance of any given length of a local shortest unique substring. Using equation (1) and knowing that the GC-content of the genome of *C. elegans *is 0.3544, one expects to find by chance alone 1.6 unique substrings of length and 10 and 20,441 unique substrings of length 11. These values agree with what we have found in the actual genome of *C. elegans *(one pair of unique substrings of length 10, and 10,509 pairs of length 11). However, again based on equation (1), the probability of finding in the human genome (GC-content = 0.4088) a unique substring of length 11 is less than 10^-100^. This is equivalent to an expected number of only 2.4 × 10^-94 ^of such unique substrings and in sharp contrast to the observed value of 215 pairs of unique substrings of this length. The same holds for mouse. Clearly, the lengths of the shortest unique substrings found in mouse and human cannot be explained by chance.

**Figure 4:**
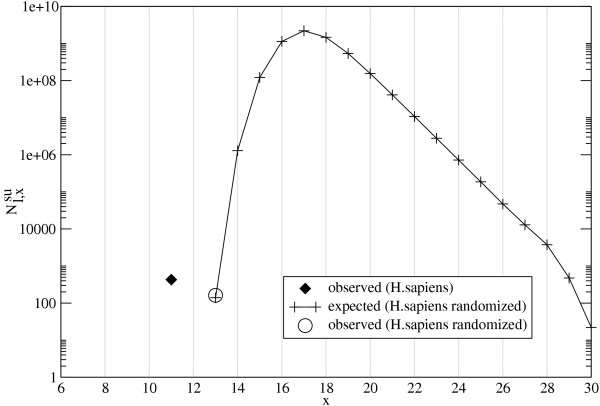
**Expected number of shustrings in the randomized human genome**. Expected number, 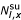, of local shortest unique substrings of length *x*. Parameters used in equation (1) are *l *= 2.84·10^9 ^and 2*p *= 0.409 and correspond to the euchromatic part of the human genome.

In addition to quantifying the probability of finding shortest unique substrings, equation (1) also allows us to estimate the lengths of unique oligos for arbitrary genomes. In the case of the human genome the length distribution is shown in Figure 4. Notice that this distribution is strongly skewed to the right with 30 being the highest length with an expected occupancy of ≥ 1.

Since equation (1) describes the length distribution of shortest unique substrings in random sequences, it is also the null-distribution for multiple, but phylogenetically unrelated, sequences. This fact can be exploited for comparative genome analysis.

### Comparing five strains of *Staphylococcus aureus*

**Figure 5:**
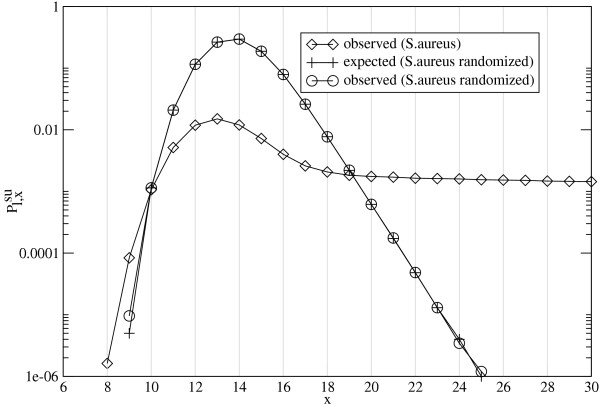
**Shustring probability distribution in five randomized strains of *Staphylococcus aureus***. Observed and expected distributions of the lengths *x *of local shortest unique substrings. Parameters *l *= 1.42·10^7 ^and 2*p *= 0.330 correspond to the combined length and average *GC*-content of five strains of *Staphylococcus aureus*.

**Figure 6:**
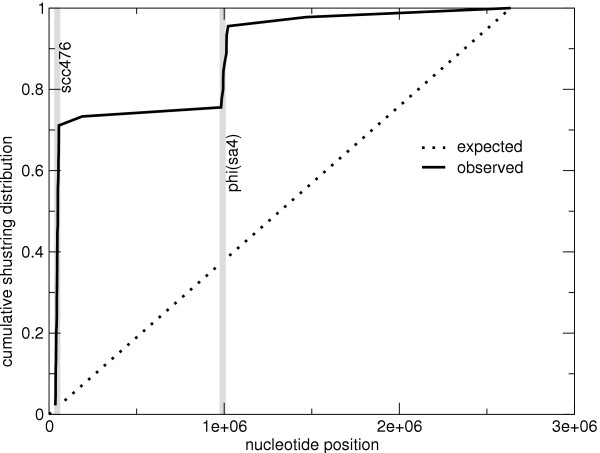
**Cumulative distribution of shustrings in *Staphylococcus aureus***. Cumulative distribution of unique substrings as a function of genome position in *S. aureus *MSSA476 when compared to strains MRSA252, MW2, Mu50, and N315 (c. f. Table 1). The steep jumps in the plot correspond to the two regions SCC_476 _(close to the origin) and ΦSa4 indicated in grey. These are known to be the sole two unique regions in the genome of MSSA476 [7]. Only local shortest unique substrings of length ≤ 10 were included in the analysis.

*Staphylococcus aureus *is a human pathogen notorious for its resistance to multiple antibiotics [7]. Five genomes of this bacterium are publicly available, which makes it one of the best characterized bacterial species. Our aim was to take strain MSSA476 [7] and identify the regions unique to its genome when compared to the four other strains available. Given the GC-content of *Staphylococcus aureus *and the combined length of the five genomes analyzed, we calculated the expected distribution of shortest unique substring lengths using equation (1). Figure 5 shows that for unrelated *S. aureus *sequences of the same combined length (14.21 × 10^6^) and composition (GC-content 0.33) as the strains investigated, the lengths of shortest unique substrings are expected to range from 9 to 27. We decided to analyze the local shortest unique substrings of length ≤ 10 in strain MSSA476 in the presence of the genomes of the four other strains. Figure 6 displays the cumulative distribution of the local shortest unique substrings of length ≤ 10 along the genome of strain MSSA476. Regions unique to MSSA476 contain a high density of such substrings and hence stand out as jumps in the cumulative plot. Figure 6 shows two such jumps. These correspond exactly to the two unique regions ΦSa4 and SCC_476 _recently annotated as the only two unique regions in MSSA476 [7].

## Discussion

The search for unique substrings has a long tradition in molecular biology. It is fundamental for many sequence-based identification techniques, and affects PCR primer design as well as the development of specific antibodies. A DNA or protein sequence of length *n *contains  substrings, which is also an upper limit for the number of unique substrings. This means that in most real world situations there is in an excess of unique substrings to choose from. Since a given unique substring remains unique upon extension, we decided to concentrate on the shortest unique substrings. In their global version they have minimal length with respect to the entire sample of sequences investigated. In contrast, their local version is defined for substrings starting from a specific position in the genome. There are of the order of *n *such local shortest unique substrings from which all the remaining unique substrings can be generated. Shortest unique substrings therefore lead to considerable space reduction when dealing with unique substrings.

Our technique for detecting such unique substrings is applicable to protein as well as to DNA sequence data. Antibodies are widely used in basic biomedical research; in addition, there is growing interest in applying them as therapeutics. A major design goal in generating antibodies to a given protein in all of these contexts is to maximize their specificity. Since the entire proteome of important biomedical model organisms, including human, is known, epitope selection might be guided not only by considerations of antigenicity, but also of uniqueness. In a preliminary study of the human proteome we found that 88% of the 27,175 human proteins we looked at contain at least one unique hexapeptide. Given that a typical epitope consists of 6 to 12 amino acids, this suggests that our method of detecting shortest unique substrings coupled with epitope prediction programs might also be useful for antibody development.

However, in this paper we have concentrated on shortest unique substrings in genomes. The fact that the length of global shortest unique substrings does not exceed 11 in autosomes of both *C. elegans *and humans is intriguing given the widely differing sizes of the two genomes and the extremely small probability of observing unique sequences of length 11 by chance in the human genome. Since the length of global shortest unique substrings remained constant after we had removed repetitive elements from the genomes, we take this as an indication that genomes contain a core of high-complexity sequences which determine the length of global shortest unique substrings. The size of this high-complexity core is apparently much less variable across metazoan genomes than raw genome size, hence the observed constancy of global shortest unique substring lengths.

These global shortest unique substrings can be used as starting points for developing signature oligos. Such oligos are widely used in biotechnology and taxonomy. A typical application in biotechnology is PCR-primers that should be unique to the target sequence. In taxonomy a recent initiative for the "Barcoding of Life"  attempts to "label" all extant species by assigning a short unique DNA-sequence to them.

The probability of finding a shortest unique substring of some length can be readily computed using equation (1). However, this equation is highly sensitive to the value of the parameter *p*, which describes the sequence composition. Hence, local variation in sequence composition will strongly affect the expected length of both local and global shortest unique substrings. This fact may also have contributed to our observation that shortest unique substrings cluster in upstream regions of genes in both the mouse and the human genomes. The euchromatic part of the human genome has an average GC-content of 0.41 (, Human genome assembly hg16), which is similar to the value of 0.42 for the mouse (, Mouse genome assembly mm4). In contrast, the global shortest unique substrings we found in humans have a GC-content of 0.59 and those found in mouse have a GC-content of 0.61. The upstream regions of genes tend to be GC-rich in human (GC-content = 0.53) as well as mouse (GC-content = 0.50), which might account for the clustering of global shortest unique substrings in these regions.

Detection of unique genomic regions is traditionally done by alignment-based approaches. However, the run-time of these algorithms depends non-linearly on the number and lengths of the input sequences and also on the degree of relatedness of the input sequences. In contrast, the scheme for detecting unique genomic regions proposed in this paper has a run time that is strictly linear in the combined lengths of the input sequences.

## Conclusion

There is currently a lot of interest in comparative genomics [8]. In many of these projects detection of regions unique to a genome is one of the first steps towards functional annotation (e. g. [7]). Given equation (1), the size distribution of shortest unique substrings in random, i.e. unrelated, sequences can be predicted. This leads to our method of detecting unique genomic regions from an arbitrary set of input sequences without the need for alignment. Its usefulness for comparative genomics is clearly demonstrated in the case of the genomes of *S. aureus*, where we could rapidly detect the two unique regions previously annotated in one strain [7] (Figure 5).

## Methods

### Detection of shortest unique substrings

Two methods borrowed from computer science were used for the detection of shortest unique substrings: suffix tree construction and hashing. Suffix trees are well described by Gusfield [4] and we follow his nomenclature. To use suffix trees for detecting unique substrings, notice that the path label of any leaf is a unique substring. The set of *shortest *unique substrings at every position can therefore be discovered by traversing the tree once and looking up the string depth of the parent node of every leaf. This value plus one is the desired length of the shortest unique string that starts at the position indicated by the leaf.

Hashing is described, for example, by Cormen *et al. *[[9], ch. [11]] and we used it for detecting global shortest unique substrings in large genomes.

Unless stated otherwise, all computations presented in this paper consider both strands of the DNA sequences concerned. Note that in this case, and due to complementarity of DNA, a single parameter (*p*) suffices to describe nucleotide composition.

### The probability distribution of local shortest unique substring lengths in nucleotide sequences

Consider a nucleotide sequence *S *and let 2*p *denote the GC-content of *S *(*p *∈ [0, 1/2]). A shortest unique substring of length *x *of this nucleotide sequence is defined as a unique substring *S*[*i*..*i *+ *x *- 1] where *S*[*i*..*i *+ *x *- 2] is not unique. We wish to derive the probability distribution of values of *x *under the assumption of random sequence composition.

We start by considering a particular substring of length *x *consisting of *k *positions occupied by either G or C each. We refer to such a substring as being of type (*x*, *k*). The probability of finding a substring of type (*x*, *k*) is

*P*_*x*,*k *_= (1/2 - *p*)^*x *- *k *^*p*^*k*^.

Assuming that *l *independent trials each having a success probability of *P*_*x*,*k *_are performed, the probability of finding a particular sequence of type (*x*, *k*) exactly once is then



This expression is only approximately valid, since the nucleotide compositions of any two overlapping subtrings are not independent. Still, from now on we assume independence. The error introduced by this assumption is negligible, if the genome size, *l*, is large compared to the length of the considered substrings (*l *>> *x*) - which is the case we have in mind. Thus, we replace the ≈-sign in the above and following expressions by = and define



For each sequence of type (*x*, *k*), there are  permutations of *k *"G|C" s and (*x *- *k*) "A|T" s. Some of these permutations occur zero times in *S*, some occur multiple times and some occur exactly once. We are interested in the latter: the number of *unique *substrings of type (*x*, *k*) is



In order to determine the number of unique substrings irrespective of their sequence composition, we need to sum over all possible values of *k*:



The number of *shortest *unique substrings of length *x*, 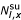, is then simply the number of unique substrings of length *x *minus the number of unique substrings of length *x *- 1. In order to see this, notice that all unique substrings of length *x *- 1 are contained in the set of unique substrings of length *x*. Those that are gained by adding the extra nucleotide are precisely the substrings that lose their uniqueness when reduced in length by one as required by the definition of shortest unique substring:



Finally, the probability of finding such a shortest unique substring of length *x*, , is the number of unique shortest substrings of length *x *divided by the genome length:



### Implementation

The search for shortest unique substrings is implemented in our program shustring (SHortest Unique subSTRING). The distribution of shortest unique substring lengths in genomic sequences as embodied in equation (1) is implemented in our program shulen. Both pieces of software are available from .

### Data

Genome sequences of the nematode (*Caenorhabditis elegans*) [5], mouse (*Mus musculus*) [10], and human (*Homo sapiens*) [6] as well as 1 kb upstream regions for genes in the genomes of human and mouse were obtained from the University of California Santa Cruz genome website at the following URLs:

1. nematode:  (version ce2)

2. mouse:  (version mm4, October 2003)

3. human:  (version hg16, July 2003)

Table 1 lists the six bacterial genomes analyzed in this study.

## Authors' contributions

B.H. designed and implemented the software, performed data analysis and contributed to the writing of the manuscript. N.P. was involved in data analysis, software testing and contributed to the writing of the manuscript. F.M. carried out the analysis of the upstream regions. T.W. conceived of the study of shortest unique substrings, derived their null distribution, and contributed to the writing of the manuscript. All authors read and approved the final manuscript.

## Supplementary Material

Additional File 1 Supplementary Material. List of Human and Mouse genes with hits to shortest unique substrings to their 1 kb upstream regions Click here for file

## Figures and Tables

**Table 1:** Bacterial genomes analyzed in this study

organism	accession number	reference	genome size
*Mycoplasma genitalium*	L43967	[11]	580,074
*Staphylococcus aureus *MRSA252	NC 002952	[7]	2,902,619
*Staphylococcus aureus *MSSA476	NC 002953	[7]	2,799,802
*Staphylococcus aureus *MW2	NC 003923	[12]	2,820,462
*Staphylococcus aureus *Mu50	NC 002758	[13]	2,878,040
*Staphylococcus aureus *N315	NC 002745	[13]	2,814,816
