# Supplementary material for: Genome comparison without alignment using shortest unique substrings
Source: BMC Bioinformatics. 2005 May 23;6:123. doi: 10.1186/1471-2105-6-123 (PMC1166540; doi:10.1186/1471-2105-6-123)
Supplement: Additional File 1 — Supplementary Material. List of Human and Mouse genes with hits to shortest unique substrings to their 1 kb upstream regions [file 1471-2105-6-123-S1.pdf]

Supplementary Material for *Genome Comparison without Alignment Using Shortest Unique Substrings*

Bernhard Haubold [a], Nora Pierstorff [b], Friedrich Möller [c], Thomas Wiehe [b*]

[a]Department of Biotechnology & Bioinformatics, University of Applied Sciences Weihenstephan, Germany, [b]Institute of Genetics, Universität zu Köln, Germany, [c]Berlin Center for Genome Based Bioinformatics and Freie Universität, Berlin, Germany

In the following two sections the human and mouse genes with hits to a shortest unique substring to their 1kb upstream region are listed and explained.

1 Human

Table 1: List of the 30 (of which 29 are distinct) shortest unique substrings ("shustrings") from human that had matches to 1kb upstream regions of annotated genes. Annotations were taken from GenBank. Notice that the genes described in entries #9 and #10 are located on opposite strands and have an overlapping upstream region. Futher, the shortest unique substrings located on the Y chromosome cluster into two overlapping groups: {#20, #21, #22}, and {#23, #24, #25}.

ID	Shustring	Accession	Position	Genbank Definition
1	`tacgcgaccgt`	NM_000437	chr1_25929337_r	Homo sapiens platelet-activating factor acetylhydrolase 2, 40kDa (PAFAH2)
2	`ttacgcgcgat`	NM_005626	chr1_29195874_r	Homo sapiens splicing factor, arginine/serine-rich 4 (SFRS4)
3	`cgacgtacgtc`	NM_013285	chr1_37489159_r	Homo sapiens nucleolar GTPase (HUMAUANTIG)
4	`ttgtcgcgtcg`	NM_006374	chr2_242768775_r	Homo sapiens serine/threonine kinase 25 (STE20 homolog, yeast) (STK25)
5	`gcgcgaacgta`	NM_004206	chr3_42584021_r	Homo sapiens SEC22 vesicle trafficking protein-like 3 (S. cerevisiae) (SEC22L3)
6	`ggcgtacgtcg`	NM_153361	chr5_43236828_f	Homo sapiens hypothetical protein MGC42105(MGC42105)
7	`gccgacgtacg`	NM_006805	chr5_137166247_r	Homo sapiens heterogeneous nuclear ribonucleoprotein A0 (HNRPA0)

*to whom correspondence should be addressed

8	tgcgcgttacg	NM_002520	chr5_170794780_f	Homo sapiens nucleophosmin (nucleolar phosphoprotein B23, numatrin) (NPM1)
9	cgcgaacggta	NM_002137	chr7_25982639_r	Homo sapiens heterogeneous nuclear ribonucleoprotein A2/B1 (HNRPA2B1)
10	cgcgaacggta	NM_016587	chr7_25982596_f	Homo sapiens chromobox homolog 3 (HP1 gamma homolog, Drosophila) (CBX3)
11	ttacgcgaccg	NM_017635	chr11_67756092_r	Homo sapiens CGI-85 protein (CGI-85)
12	cgcgacgtacg	NM_015416	chr12_49727399_f	Homo sapiens cervical cancer 1 protooncogene (HCCR1)
13	cgacgcgaata	NM_015646	chr12_67289978_f	Homo sapiens RAP1B, member of RAS oncogene family (RAP1B)
14	acgtaccgtcg	NM_052903	chr15_20380181_f	Homo sapiens tubulin, gamma complex associated protein 5 (TUBGCP5)
15	tcgattcgcgg	NM_032724	chr18_58338821_f	This record was temporarily removed by RefSeq staff for additional review
16	cgcgcgacatt	NM_033257	chr22_18682163_r	Homo sapiens DiGeorge syndrome critical region gene 6-like (DGCR6L)
17	cggacgatcgg	NM_025204	chr22_48805388_f	Homo sapiens hypothetical protein PP2447 (PP2447)
18	tacgtcgcgag	NM_006649	chrX_127744782_f	Homo sapiens UTP14, U3 small nucleolar ribonucleoprotein, homolog A (yeast) (UTP14A)
19	acatcgcgcgaa	NM_004660	chrY_14024442_f	Homo sapiens DEAD (Asp-Glu-Ala-Asp) box polypeptide 3, Y-linked (DDX3Y)
20	catcgcgcgaat	NM_004660	chrY_14024442_f	Homo sapiens DEAD (Asp-Glu-Ala-Asp) box polypeptide 3, Y-linked (DDX3Y)
21	atcgcgcgaatt	NM_004660	chrY_14024442_f	Homo sapiens DEAD (Asp-Glu-Ala-Asp) box polypeptide 3, Y-linked (DDX3Y)
22	tcgcgcgaattt	NM_004660	chrY_14024442_f	Homo sapiens DEAD (Asp-Glu-Ala-Asp) box polypeptide 3, Y-linked (DDX3Y)
23	taaacgaacgcg	NM_004660	chrY_14024442_f	Homo sapiens DEAD (Asp-Glu-Ala-Asp) box polypeptide 3, Y-linked (DDX3Y)
24	aacgaacgcgat	NM_004660	chrY_14024442_f	Homo sapiens DEAD (Asp-Glu-Ala-Asp) box polypeptide 3, Y-linked (DDX3Y)

25	cgaacgcgattt	NM_004660	chrY_14024442_f	Homo sapiens DEAD (Asp-Glu-Ala-Asp) box polypeptide 3, Y-linked (DDX3Y)
26	cattattcgcgt	NM_004202	chrY_14823112_f	Homo sapiens thymosin, beta 4, Y-linked (TMSB4Y)
27	cgccggcgatat	NM_004653	chrY_20801994_r	Homo sapiens Jumonji, AT rich interactive domain 1D (RBP2-like) (JARID1D)
28	gttatgcggtcg	NM_031932	chrY_20134487_r	This record was temporarily removed by RefSeq staff for additional review.
29	tacggagcgacc	NM_173700	chrY_20560224_r	Homo sapiens hypothetical protein FLJ39821 (FLJ39821)
30	taccgcgacata	NM_144971	chrY_23299190_f	This record was temporarily removed by RefSeq staff for additional review.

2 Mouse

Table 2: List of the 22 shortest unique substrings ("shustrings") from mouse that had matches to 1kb upstream regions of annotated genes. Annotations were taken from GenBank. Notice that sequence #9 has not been placed on the genome in the NCBI-Build of the Mouse Genome (build 32 version 1), hence the qualification of the position as "random". In the genome build used for the detection of the shortest unique substrings this particular substring occurred on chromosome 19.

ID	Shustring	Accession	Position	Genbank Definition
1	cgtatcgcgcc	NM_022019	chr1_187670232_f	Mus musculus dual specificity phosphatase 10 (Dusp10)
2	taaatcgcgcg	NM_152947	chr2_144403782_r	Mus musculus zinc finger protein 339 (Zfp339)
3	gtcgacgctcg	NM_028125	chr2_181307449_r	Mus musculus BTB (POZ) domain containing 4 (Btbd4)
4	cgtacgcgacg	NM_026578	chr3_133463660_r	Mus musculus nucleolar protein family A, member 1 (H/ACA small nucleolar RNPs) (Nola1)
5	tcgcgaacgta	NM_021535	chr4_40805423_r	Mus musculus smu-1 suppressor of mec-8 and unc-52 homolog (C. elegans) (Smu1)
6	ctcgcgtcgac	NM_053124	chr4_145081130_f	Mus musculus SWI/SNF related, matrix associated, actin dependent regulator of chromatin, subfamily a, member 5 (Smarca5)
7	ccgatcgcgtt	NM_172998	chr5_117152145_f	Mus musculus RIKEN cDNA B830028P19 gene (B830028P19Rik)
8	ttagcgcgtcg	NM_033572	chr5_133175102_f	Mus musculus Williams-Beuren syndrome chromosome region 16 homolog (human) (Wbscr16)
9	tcgacgcgcta	NM_027732	chr7_random_15462377_r	Mus musculus doublesex and mab-3 related transcription factor like family C2 (Dmrtc2)

10	cgccgaatacg	NM_177468	chr7_39912224_r	Mus musculus DNA segment, Chr 7, ERATO Doi 413, expressed (D7Ertd413e)
11	tccgattcgcg	NM_133755	chr7_131926212_r	Mus musculus tubulin, gamma complex associated protein 2 (Tubgcp2)
12	atacgcgacga	NM_008578	chr8_70336585_f	Mus musculus myocyte enhancer factor 2B (Mef2b)
13	tcgaacgaccg	NM_009864	chr8_108133432_f	Mus musculus cadherin 1 (Cdh1)
14	cgtatcgcgga	NM_178374	chr9_59055636_f	Mus musculus RNA, U transporter 1 (Rnut1)
15	cgcgacgttag	NM_153098	chr9_81196302_f	Mus musculus CD109 antigen (Cd109)
16	acgacgttcgc	NM_181411	chr11_20640159_r	Mus musculus RIKEN cDNA 9130023F12 gene (9130023F12Rik)
17	taacgtcgcga	NM_030238	chr12_106168459_f	Mus musculus dynein, cytoplasmic, heavy chain 1 (Dnchc1)
18	tcgtcgcgaat	NM_025815	chr15_92413205_r	Mus musculus copine VIII (Cpne8)
19	cgaccgtcgca	NM_053181	chr16_13464863_r	Mus musculus expressed sequence AA415817 (AA415817)
20	cttcgacgcga	NM_011949	chr16_16588084_f	Mus musculus mitogen activated protein kinase 1 (Mapk1)
21	atcgcgttcgg	NM_007522	chr19_6933099_f	Mus musculus Bcl-associated death promoter (Bad)
22	atttcgcgcga	NM_172307	chrX_145621642_r	Mus musculus membrane-bound transcription factor protease, site 2 (Mbtps2)
